# Structural Characterisation of Ligand-Binding Determinants in Human Lung Surfactant Protein D: Influence of Asp325

**DOI:** 10.1016/j.jmb.2009.09.057

**Published:** 2009-12-11

**Authors:** A.K. Shrive, C. Martin, I. Burns, J.M. Paterson, J.D. Martin, J.P. Townsend, P. Waters, H.W. Clark, U. Kishore, K.B.M. Reid, T.J. Greenhough

**Affiliations:** 1 Research Institute of Science and Technology in Medicine, and School of Life Sciences, Keele University, Staffordshire ST5 5BG, UK; 2 MRC Immunochemistry Unit, Department of Biochemistry, University of Oxford, South Parks Road, Oxford OX1 3QU, UK; 3 Weatherall Institute of Molecular Medicine, John Radcliffe Hospital, University of Oxford, Headington, Oxford OX3 9DS, UK

**Keywords:** SP-D, lung surfactant protein D, hSP-D, human lung surfactant protein D, rfhSP-D, recombinant fragment of human lung surfactant protein D, MBP, mannose-binding protein, CRD, carbohydrate recognition domain, MPD, 2,4-methylpentane diol, PI, phosphatidylinositol, IP, inositol 1-monophosphate, PDB, Protein Data Bank, lung surfactant protein, crystal structure, collectin, carbohydrate recognition

## Abstract

The crystal structures of a biologically and therapeutically active recombinant homotrimeric fragment of human lung surfactant protein D with a series of bound ligands have been determined. While the structures reveal various different binding modes, all utilise a similarly positioned pair of mannose-type O3′ and O4′ hydroxyls with no direct interaction between any non-terminal sugar and protein. The orientation, position, and interactions of the bound terminal sugar depend on the sugar itself, the presence and form of glycosidic linkage, and the environment in the crystal, which, via Asp325, places stereochemical and electronic constraints, different for the three different subunits in the homotrimer, on the ligand-binding site. As a direct consequence of this influence, the other binding-pocket flanking residue, Arg343, exhibits variable conformation and variable interactions with bound ligand and leaves open to question which orientation of terminal mannobiose, and of other terminal disaccharides, may be present in extended physiological ligands. The combined structural evidence shows that there is significant flexibility in recognition; that Asp325, in addition to Arg343, is an important determinant of ligand selectivity, recognition, and binding; and that differences in crystal contact interfaces exert, through Asp325, significant influence on preferred binding modes.

## Introduction

Human lung surfactant protein D (hSP-D) exerts its biological role through recognition of an array of carbohydrate present in the surface lipopolysaccharides and phospholipids,^1,2^ peptidoglycans and glycosaminoglycans^3,4^ of a wide range of microbial targets, such as viruses, bacteria, yeasts, and fungi.^5–7^ Interaction of hSP-D with these pathogens promotes microbial aggregation and enhanced uptake and killing by neutrophils and alveolar macrophages.^7^ Recent binding studies have also suggested a role for SP-D in the recognition and clearance of foreign and host nucleic acids, and apoptotic cells, in the lung, thus down-regulating inflammation and minimising autoantibody generation.^8–10^ Although predominantly characterised in the lung, hSP-D has also been shown to be expressed at various mucosal sites.^11^ Structurally,^12^ SP-D adopts a tetrameric cruciform-like structure, with four arms of equal length. Ligand recognition occurs in carbohydrate recognition domains (CRDs), which are spatially separated, in a trimeric configuration, at the end of triple-helical collagenous stalks linked to the CRDs through short α-helical coiled-coil neck domains.

Pulmonary SP-D specifically binds to phosphatidylinositol (PI),^2^ which accounts for 3% (w/w) of surfactant. While previous reports are unclear as to whether this binding involves the neck, the CRD, or both,^1,2,13^ inositol phosphate and *myo*-inositol have recently been shown to bind to the principal CRD calcium ion, suggesting that the CRD mediates PI recognition.^14^ The interaction of SP-D with phospholipids influences surfactant homeostasis and alveolar cell morphology, with SP-D-deficient mice exhibiting an accumulation of lung surfactant lipids and abnormalities in the morphology of alveolar type II cells and macrophages.^15^ It has been suggested^16^ that PI binding may inhibit interaction of SP-D with microorganisms *in vivo*, although SP-D-induced microbial aggregation in the airways, which contributes *in vivo* to host defence, does not appear to be affected by this interaction.

Current structural knowledge indicates that pathogen recognition in the lungs is mediated by CRD Ca-dependent binding of terminal monosaccharide residues^14,17–19^ characteristic of bacterial and fungal cell surfaces, although modelling studies predict that binding can also occur through internal (non-terminal) residues in glucosyl trisaccharides.^20^ Recent structural studies of glucosyl trisaccharides have demonstrated that, in addition to terminal sugar glc1 binding by the principal CRD calcium ion, a phenylalanine residue proximal to the carbohydrate-binding groove interacts directly with glc3 and is critical for simple maltoside and maltotriose binding.^18^ It has been suggested that the fairly nonspecific monosaccharide binding and the geometrical relationship between the multiple CRDs may explain the ability of the collectins to recognise and bind to the surface patterns of carbohydrate presented by pathogenic microorganisms and thus discriminate between self and non-self.^21^

**Fig. 1:**
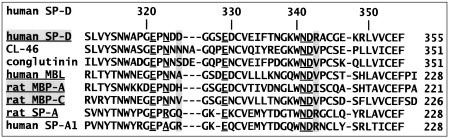
C-terminal sequences of selected collectins (hSP-D numbering). Known neck/CRD structures are underlined, and reported ligand-bound structures are highlighted. Residues that bind to both calcium and (except SP-A 323 and 342) ligand are underlined, and those that flank the ligand-binding site (SP-D 325 and 343) are highlighted.

The Ca-dependent carbohydrate-binding pocket in the collectins is highly conserved in terms of the residues that coordinate to both the calcium ion and the ligand through a pair of hydroxyls positioned in a manner similar to the mannose-type O3′ and O4′ equatorial pair (see Fig. 1). The SP-A residue that replaces Asn323 (hSP-D numbering) is oriented such that the main-chain carbonyl is placed in the position of Asn323 OD1 in other collectins.^22^ The roles of both Arg343^14,17,23,24^ and Asp325^17,25^ in SP-D ligand selectivity and specificity have been investigated by structural, modelling, and mutagenesis studies. Elegant work by Weiss *et al.* on ligand-bound structures of rat MBP^26–28^ suggests that the highly variable residue equivalent to hSP-D 325 on one side of the binding site is a major determinant of bound ligand orientation in MBP. Insertion of the CL-43 Arg-Ala-Lys (RAK) motif to immediately precede Asp325 in hSP-D results in enhanced interactions with influenza A virus and saccharide-binding characteristics that more closely resemble CL-43.^29^ On the opposite side of the binding site, the residue equivalent to Arg343 is conserved as Arg or Lys in all known SP-D sequences, and its role in PI recognition and binding,^14^ in discrimination between glucose and GlcNAc,^23^ in recognition of multivalent microbial ligands,^24^ and in the selection, recognition, and binding of physiological ligands in general^17^ has been highlighted. There is, however, no clear explanation of the structurally characterised variability of orientation or of the relative affinity for the variety of ligands recognised by the collectins.

A recombinant homotrimeric form of truncated hSP-D (rfhSP-D) expressed in *Escherichia coli*, composed of the neck region plus three CRDs, has been shown to have therapeutic effects in murine models of pulmonary hypersensitivity and infection induced by an opportunistic fungus, *Aspergillus fumigatus*.^30–32^ Although rfhSP-D constitutes only a relatively small fragment (60 kDa) of the entire natural hSP-D molecule (540 kDa), it appears to retain significant biological activity both *in vitro* and *in vivo*.^32–35^ The structure of rfhSP-D has been reported to 2.3 Å^36^ as well as high-resolution structures of rfhSP-D (1.4 Å) and maltose-bound rfhSP-D (1.6 Å).^17^ More recent structural work on a recombinant neck + CRD hSP-D trimer has included complexes with maltotriose, *p*-nitrophenyl maltoside, *myo*-inositol, inositol-1-phosphate, heptoses, α-Me-man, and α1-2 mannobiose1.^4,18,19,24^ Inositol-1-phosphate binds via O4′ and O5′ in a similar manner to the standard mannose-type binding through the adjacent equatorial hydroxyls O3′ and O4′,^14^ and l,d-heptose has been shown to bind preferentially through the O6′ and O7′ hydroxyls of the glycerol side chain rather than through the standard O3′ and O4′ of the pyranose ring.^19^ In both cases, the coordinated hydroxyls are positioned and bound in a manner almost identical with the mannose-type equatorial hydroxyl pair O3′ and O4′, which bind in the remainder of the array of structurally characterised hSP-D–ligand complexes.^14,17–19,24^

Recent binding studies based on competition with the SP-D–mannan interaction on BIAcore chip have suggested novel ligands for hSP-D. These ligands include bacterial and host-genome nucleic acids and their components such as d-ribose and (deoxy)ribonucleotide triphosphates,^9,10^ peptidoglycan components such as *N*-acetyl muramic acid,^3^ and the glycosaminoglycans of proteoglycans such as the GalNAc-4-sulfate contained in the dermatan sulfate component of decorin.^4^ Due to our failure to introduce these ligands into rfhSP-D crystals, either by soaking or co-crystallisation, and to further investigate the binding of mono- and disaccharides, we have undertaken an in-depth structural analysis of a series of rfhSP-D–ligand complexes including manα1–2man (characteristic of the branch repeating unit of mannan), manα1–4man (providing a direct comparison with manα1–2man and with maltose,^17^ the favoured ligand of hSP-D), and galactose. For consistency of interpretation, we have redetermined the structure of the physiologically important inositol 1-monophosphate (IP) complex^14^ to higher resolution for a more complete data set [99% complete to 1.75 Å here; 92% complete to 1.89 Å in Protein Data Bank (PDB) file 2ork^14^].

## Results

### Overall structures

**Fig. 2:**
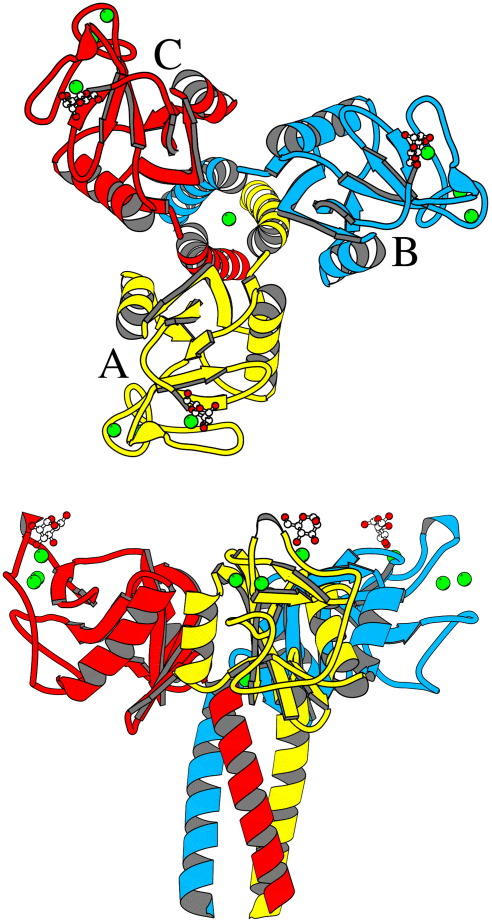
The manα1–2man-bound rfhSP-D trimer showing the bound manα1–2man (only the terminal mannose man1 is visible in the electron density) and the calcium ions (green spheres). (a) Viewed down the molecular 3-fold axis. (b) Viewed perpendicular to the molecular 3-fold axis.

**Fig. 3:**
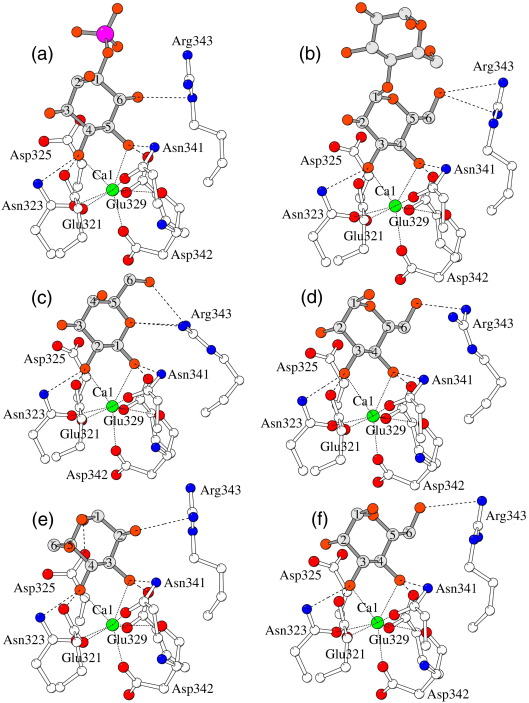
The coordination of the calcium ion Ca1 and the bound ligands in selected subunits of the rfhSP-D–ligand complexes. (a) Chain B of the inositol phosphate structure. (b) Chain A, maltose. (c) Chain A, galactose. (d) Chain A, manα1–2man. (e) Chain B, manα1–4man. (f) Chain A, manα1–4man.

**Fig. 4:**
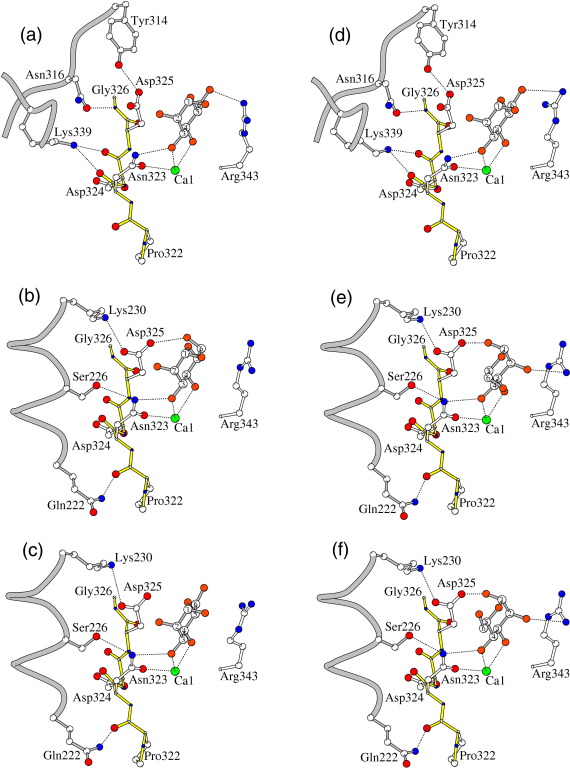
The influence of the variable crystal contacts on the orientation and interactions of bound mannobiose and Arg343. (a–c) manα1–2man chains A, B, and C, respectively. (d–f) manα1–4man chains A, B, and C, respectively. In all figures, the symmetry contact protein molecule and the bound terminal mannose are represented in grey, with selected residues of the hSP-D monomer represented by yellow main-chain bonds and white side-chain bonds. Coordination (to Ca1 and ligand) distances are given in Table 1 with symmetry contact distances in Table 2. The crystal contacts with chains B and C, in both structures, are very similar but not identical. Of the six subunit structures, the only pairs with similar ligand orientation and similar ligand non-bonded contacts are chains B and C in manα1–4man and chain A in both manα1–2man and manα1–4man, although in the latter case, the two orientations of the Arg343 side chain are significantly different.

**Fig. 5:**
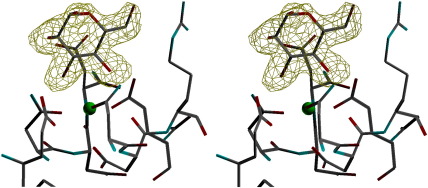
Stereo view of the electron density in the chain A calcium and ligand-binding site of the α1-4 mannobiose-bound structure. The difference electron density map (*F*_o_ − *F*_c_) is calculated from the observed data (*F*_o_) and the model (*F*_c_) prior to adding ligand into the model but after location and refinement of water molecules (none of which moved into the ligand-binding site on refinement). The map, calculated at 1.65 Å resolution, is solvent flattened and contoured at 2.5 r.m.s.d.

The overall structures of rfhSP-D reveal the trimeric aggregate with three spatially separated C-terminal globular CRD domains (residues 236–355) and an extended α-helical coiled-coil neck region (residues 203–235) (Fig. 2). The first few residues of the neck region and the 24-residue N-terminal collagen tail are not visible in the electron density map. While the structures reveal various different ligand-binding modes, all utilise a similarly positioned pair of mannose-type O3′ and O4′ hydroxyls (Figs. 3–5 and Table 1) with no direct interaction between any non-terminal sugar and protein. The calcium ions reported in the rfhSP-D structure, three per CRD plus Ca4, located on the trimeric axis in a pore at the bottom of the funnel formed by the three CRDs and close to the neck–CRD interface,^17^ are present in all the ligand-bound structures presented here. Within the molecular trimer, the calcium ion Ca4 is coordinated by all three Glu232, each of which also interacts with Lys246 in the same chain. These exposed lysines form a positively charged surface around the trimeric axis at the bottom of the central pore. In the galactose-bound structures, there is evidence of a minor conformation of Glu232 in chain B, which allows the side chain (OE1) to interact with the asymmetric tyrosine Tyr228 in chain C rather than Lys246 in chain B.

In this crystal form, the ligand-binding site flanking residue Asp325 in all three chains forms a crystal contact to other trimers, with a broad contact region covering residues 322–326 (see Table 2 and Fig. 4). In chains B and C, the crystal contact interfaces are very similar, but not identical, and include a charge–charge interaction between Asp325 OD1 and Lys230 NZ in a neighbouring trimer, with, over the four structures, distances of 2.77–2.99 Å for chain B and 2.85–3.24 Å for chain C. The only other significant specific contacts in this region for chains B and C are hydrogen bonds between the main-chain O of Pro322 and Gln222 NE2 (2.97–3.01 Å for chain B and 2.83–3.11 Å for chain C) and between Asn323 ND2 and Ser226 OG in the neighbouring molecule (averaging 2.93 Å for chain B and 3.07 Å for chain C). The contact to Asn323 ND2, which coordinates directly to ligand, rotates the side chain, moving ND2 towards the contacting serine and away from the ligand-binding pocket as compared to chain A, resulting in a small but significant shift in the orientation of the bound ligand (Fig. 4).

In chain A, the 322–326 crystal contact interface, which is entirely different to that in chains B and C (see Table 2 and Fig. 4), results in the chain A ligand-binding site being more restricted than those in the other two chains. The Asp325 crystal contact is in the form of a hydrogen bond of 2.58–2.77 Å between Asp325 OD1 and Tyr314 OH in chain B of a neighbouring trimer, and the orientation of the side chain of Asp325 in chain A is significantly different to that in chains B and C. Other significant crystal contacts at this interface include the main-chain carbonyls of Asp324, whose side chain coordinates to Ca2,^17^ the ligand-binding Asn323 interacting with Lys339 NZ in the neighbouring molecule (2.91–3.30 Å and 2.68–2.95 Å, respectively), and the main-chain N of Gly326 interacting with Asn316 OD1 (2.82–2.91 Å).

The crystal contacts through Asp325 affect the position and orientation of bound ligand, which, in turn, influences the side chain of Arg343 that flanks the binding pocket on the opposite side to Asp325. The orientation of the side chain of Arg343 varies both within and across the various ligand-bound structures (as was reported for the maltose-bound structure),^17^ particularly within the manα1–2man and manα1–4man structures (Figs. 3 and 4). The interactions between the ligand and Arg343 also vary within and across the four structures, but in all cases, they involve only the terminal sugar. The intrachain interaction between Arg343 and Glu333 OE2 reported for the maltose-bound structure is also present in the structures reported here, but both the strength and the nature of the interaction vary with the orientation of Arg343.

### Mannobiose binding

While both manα1–2man and manα1–4man coordinate to the CRD calcium ion Ca1 via O3′ and O4′ of the “terminal” sugar man1, the orientation of the terminal sugar varies and the second mannose in the ligand is not visible in any of the CRDs (Fig. 5). The water-mediated crystal contact, which results in the orientation of the second sugar being defined in chain A of the maltose-bound structure,^17^ is not present in either of the mannobiose structures, which have the same space group with closely similar unit cell parameters. In the manα1–2man complex, the terminal mannose man1 displays the standard orientation in all three CRDs, with the O3′ atom of man1 interacting with Glu321 OE2 and with ND2 of Asn323 and the O4′ atom interacting with OE2 of Glu329 and with ND2 of Asn341 (see Figs. 3–5 and Table 1). In chain A, there is an additional 2.87 Å interaction of O6′ with NH1 of Arg343, while in chain B, Arg343 is oriented differently and O6′, though poorly defined in the electron density, is rotated to interact with OD2 of Asp325 (2.85 Å), which lies on the opposite side of the ligand-binding pocket to Arg343. In the remaining subunit (chain C), where the crystal contact made by Asp325 to the neighbouring Lys230 is particularly weak (3.24 Å), the orientation of Arg343 differs markedly from that in chains A and B, and the bound ligand makes no additional direct contacts with protein. The head group of Arg343 is, however, aligned parallel with the C5–C6′–O6′ group of man1 at a distance of 3.8 Å. Glu333 OE2 interacts with Arg343 NH2 and NE in chain A, with Arg343 NH1 in chain B and Arg343 NE in chain C.

The manα1–4man complex structure displays two orientations of the terminal mannose. In the chain A CRD, the terminal mannose displays the standard orientation, similar to the manα1–2man and maltose structures, with the Arg343 head group aligned parallel with the mannose C5–C6′–O6′ group with O6′ making contacts of 3.34 Å with Arg343 NH2 and 3.19 Å with Arg343 CZ. In chain C, the terminal sugar is rotated by 180° with O3′ and O4′ interchanged relative to the standard orientation seen in the manα1–2man structure (Fig. 4). The O4′ atom of man1 thus interacts with Glu321 OE2 and Asn323 ND2, and the O3′ atom interacts with OE2 of Glu329 and ND2 of Asn341 (Table 1). There are contacts of 3.29 Å between O2′ and Arg343 NH1 and 3.22 Å between O1′ and Asp325 OD2. In chain B, there is evidence that both orientations of the terminal mannose are present, although the alternative orientation is much better defined in the electron density, revealing contacts of 3.20 Å between O2′ and Arg343 NH1 and 3.39 Å between O1′ and Asp325 OD2. The conformation of Arg343 itself varies between the subunits, with that in chain A being less well defined and markedly different to that in chains B and C. The interaction between Glu333 OE2 and Arg343 NH1 is absent in chain A.

### Galactose binding

The rfhSP-d-galactose structure crystals reveals that galactose binds in a single orientation in the CRD site via O1′ and O2′, which have a similar equatorial configuration to the mannose-type O3′ and O4′ but with O1′ coordinated to Asn341 and Glu329 and O2′ coordinated to Asn323 and Glu321 (see Fig. 3 and Table 1). The bound galactose, which is very well defined in the electron density, is rotated 180° compared to the terminal glc sugar of maltose so that the opposite sugar ring face is proximal to the Arg343 side of the pocket. It is also rotated 180° about the plane of the ring, resulting in an orientation in the ligand-binding site, which spatially mimics the orientation of bound maltose. Although C6′ is in a different position, O6′ in both structures is in a very similar position. The bound ligand interacts with Arg343 through a network of contacts (2.92–3.24 Å) between galactose O5 and O6′ and Arg343 NH1 and NH2. These interactions are similar in chains B and C (average, 3.02 Å), as is the orientation of Arg343 and Asp325, but are consistently longer in chain A (average, 3.19 Å) where the conformation of Arg343 and Asp325 differs from that in the other two chains. There are no direct contacts between galactose and Asp325. There are also interactions between O3′ and O4′ and water molecules, and in chain A, O3′ interacts through a water molecule with Gln281 OG1 in chain B of a symmetry-related protein trimer.

### Phospholipid-type binding

The overall features of the structure of the rfhSP-D–inositol monophosphate complex are essentially similar to those of the structure reported by Crouch *et al.*,^14^ although the structure reported here has been refined to significantly higher resolution and with a more complete data set to that resolution [99% complete to 1.75 Å, 65,000 independent reflections here (see Table 3); 92% complete to 1.89 Å, 44,000 independent reflections in PDB file 2ork^14^]. In addition, the central calcium ion Ca4 is absent in the earlier structure, resulting in asymmetry in the Glu232–Lys246 interactions, with Glu232 in chain B repositioned to interact with the side chain of the asymmetric tyrosine TyrC228.^36^ As reported by Crouch *et al.*,^14^ the electron density reveals bound IP in only two of the three CRDs with IP being absent in chain A. Binding is the same in CRD B and C, IP coordinating to the principal CRD calcium via O4′ and O5′ in a similar manner to the standard mannose-type binding through the adjacent equatorial hydroxyls O3′ and O4′ (see Table 1). There is also an interaction between IP O6′ and NE of Arg343 (2.93 Å in chain B, 3.20 Å in chain C) and, via a water molecule, between O6′ and Asp325 OD2. The variation between chains B and C in the Asp325 and Asn323 crystal contacts is thus reflected in slight variations in the IP orientation, which is, in turn, accompanied by different orientations of Arg343 and different interactions between ligand and Arg343. This difference is also evident in the Glu333 OE2–Arg343 NH1 interaction, which is weaker in chain C. The phosphate moiety is clearly visible in the electron density map although the fine detail of the oxygens is not clear. There are no close contacts (< 4 Å) between the phosphate moiety and protein.

## Discussion

The additional calcium ion Ca4 located on the trimeric axis at the neck–CRD interface, as first observed in the rfhSP-D structure without bound ligand,^17^ is present in all the ligand-bound structures reported here and is always observed when calcium sufficient to maintain a calcium concentration of 2 mM or more within the crystal is included in the cryobuffer.^17^ In the presence of Ca4, the three interactions between Glu232 and Lys246 are symmetric, while in the absence of Ca4, GluB232 undergoes a conformational change to interact with the asymmetric tyrosine TyrC228 rather than LysB246. This conformation of GluB232 was reported in the maltose-bound structure, which has the central calcium site depleted, and is present as a minor conformation in the galactose structure here and in the ligand-free calcium-bound structure.^17^

The structures are all consistent with all other known hSP-D ligand-bound structures in that a hydroxyl pair positioned similarly to mannose-type O3′ and O4′ hydroxyls is required for binding, with, for simple ligands, the bound sugar adopting an orientation to enable this. The direct interactions between protein and ligand at the primary ligand-binding site Ca1 are, in virtually all reported structures, restricted to the terminal sugar, with minimal conformational change upon binding and the topology of the CRD site held ready for the ligand.^17^ The exceptions are the previously reported manα1–2man complex^24^ and simple maltoside and maltotriose binding^18^ where interaction between the third sugar ring and Phe335 is implicated.

While the opportunity for the second sugar man2 in chain A of the mannobiose structures to make a crystal contact through a water molecule, as observed in the maltose-bound structure,^17^ is there, it is not taken. The orientation of man2 is thus not constrained in any of the CRDs in either structure, and as expected, there is no coherent electron density that can be assigned to this mannose. This contrasts with the manα1–2man complex structure of Crouch *et al.*,^24^ in the same crystal form as that described here, where the second mannose in chain B, and only chain B, is clearly defined.

While the ligand-binding residues are remarkably conserved in the collectins (see Fig. 1), variability in other binding determinants within the binding pocket is evident throughout the family, including the two residues that flank the entrance to the pocket, Arg343 and Asp325 in hSP-D. A considerable body of work indicates that Arg343 plays a key role in ligand selectivity, specificity, and binding,^14,17,23,24^ but evidence of the influence of Asp325 is relatively uncommon.^17,25^ In rat MBP, it has been proposed that the bound sugar orientation is determined by favourable long-range van der Waals interaction with the residues equivalent to the Asp325-Arg343 pair in human SP-D. These residues are His189 and Ile207 in rat MBP-A and Val194 and Val212 in rat MBP-C^26–28^ (see Fig. 1). The structures reported here suggest that the medium- to long-range weak interactions between the sugar and the protein, involving primarily both Arg343 and Asp325, determine the position and orientation of the ligand and the relative positions of the O3′ and O4′ equivalent hydroxyls and exert significant influence on specificity. In a physiological setting, it must be assumed that binding of the terminal sugar coupled with the weak interactions is sufficient to preferentially select one particular disposition of the ligand (as observed here in galactose, manα1–2man, and IP), but that variation between different ligands with the same terminal configuration (e.g., manα1–4man and manα1–2man here) is observed due to the linkage and nature of the non-terminal carbohydrate units and their interactions with the protein. In some cases (e.g., manα1–4man here), multiple orientations of the same simple ligand are observed, but while it may be possible that selectivity fails to distinguish between the different possibilities, it seems more probable that the crystal environment itself is the determining factor.

In all the known hSP-D structures, all based on the same crystal form as first reported by Håkansson *et al.*,^36^ the crystal contacts impinge directly but variably on the binding site configuration in all three CRDs. The resulting variable orientation of Asp325 as determined by its interactions with symmetry-related molecules, which differ for the three chains, is clearly influential in determining the variation in ligand orientation, particularly for disaccharides and higher-order saccharides, and hence, through the interactions of the bound sugar with Arg343, the variable orientations of Arg343 itself. This is further evidenced by the structure without bound ligand,^17^ where the conformation of Arg343 is similar in all three chains. Thus, despite the lack of close (< 3.4 Å) interaction between the ligand and Asp325 except in one chain of each of the mannobiose structures, the interaction of the bound ligand with Arg343 appears to be significantly influenced by Asp325. However, although the structural data clearly indicate that Asp325 is an important ligand-binding determinant, the crystal influence, which constrains the side chain in a non-physiological orientation in each chain, precludes a full interpretation of its role and leaves open to question which orientation of terminal mannobiose, and of other terminal disaccharides, may be present in extended physiological ligands. Close contact of Asp325 with bound ligand, for example, with mannose O2′ in the standard mannose orientation (see Fig. 4a–d), could be achieved through only small torsional rotations of the Asp325 side chain. This interaction is reported to be present in the manα1–2man complex of Crouch *et al.*,^24^ calculated distances being 3.35–4.05 Å across the three chains. The freedom to orient the Asp325 side chain to interact with incoming and bound ligand may result in significantly different modes of ligand binding. The adjacent glycine pair Gly326 and Gly327 (with Gly326 also constrained by a crystal contact in chain A) suggests significant flexibility in the position and orientation of Asp325 in a physiological setting.

In the rfhSP-D IP structure, a symmetry-related trimer in close proximity to the CRD site in the subunit without bound ligand (chain A) may be partially occupying, or preventing entry to, the IP binding pocket, although maltose binding in the equivalent site has been observed to utilise this contact to bind the non-terminal glucose via a water molecule.^17^ Placing IP in this chain in a similar orientation as is observed in the other two CRDs results in a clash between the phosphate and the nearby symmetry-related rfhSP-D trimer. Although the phosphate could rotate away to alleviate these contacts, the defined phosphates in the IP bound to chains B and C, in a similar conformation and free of crystal contact restraints in both chains, suggest that there is a particular preferred stereochemistry, not achievable in chain A, of IP when bound to hSP-D. This conformation, and the binding geometry itself, would allow PI binding, with no steric hindrance between protein and ligand, to be achieved by superposition of the terminal IP on the bound IP determined here. It has been suggested that the positive charge associated with Arg343 may contribute to the binding of hSP-D to negatively charged ligands such as PI.^23^ More recent binding studies^14^ show, however, that both the human SP-D CRD and the R343A mutant bind poorly to PI as compared with rat SP-D, which has lysine at position 343. The human R343K mutant greatly enhances PI binding while the reciprocal mutation in the recombinant rat protein (K343R) decreases binding. This suggests that in the human protein, PI recognition depends largely on interactions with the inositol moiety.^14^ Indeed, the structure reported here shows that the phosphate group does not interact with Arg343, which is proximal and available, or with any other region of the protein, providing further evidence of the dominance of inositol recognition and a single, preferred IP stereochemistry in the hSP-D/IP complex. While there is no significant direct contact between bound IP and Asp325, the variable crystal contacts result in differing IP–Arg343 interactions in chains B and C and differing Arg343 side-chain orientation, although to a lesser extent than observed for the mannobiose structures.

The influence of the variable crystal contacts appears to be much reduced for the simple sugar galactose as compared to mannobiose and, to a lesser extent, IP, with the orientation of Arg343 and the interactions between galactose and Arg343 being similar in all three chains. While the structural results suggest a relatively high affinity for galactose, competitive ligand-binding studies^13,37^ show that hSP-D has ligand specificity for glucose and mannose type-ligands in preference to galactose. Presumably, despite the network of interactions between O6′ and O5′ and Arg343 NH1 and NH2, not present in any of the other structures, the 180° reversal of the sugar ring presents a much less favourable electronic configuration to the binding pocket.

In the mannobiose structures, the terminal mannose displays the standard orientation in all three CRDs of the manα1–2man complex but variable orientation for manα1–4man (see Fig. 4). The orientation in manα1–2man is similar to that reported for the terminal α-glucose of maltose-bound rfhSP-D^17^ and the maltotriose and *p*-nitrophenyl maltoside structures^18^ and for α-mannose of both manα1-3man and an oligosaccharide complexed with the rat MBP-A CRD.^26,27^ The reason for the lack of definition of man2 in chain B of the manα1–2man structure reported here, as opposed to the clearly defined sugar in this chain in the previous determination,^24^ is not clear, although this may be a result of ligand molarity (10 mM final concentration here, not known for the previous structure), resolution (2.25 Å here, 1.9 Å for the previous structure), or the different buffers used for crystallisation. This structural difference, as well as the variability of ligand position and orientation within both structures, does, however, serve to reinforce the influence of the variable crystal contacts through Asp325, since the contacts in chain B result in significant shifts in bound ligand, and Arg343 oriented differently, compared to the other two chains. This presumably gives rise to the reported interaction of man2 with Arg343 in, and only in, this chain of the previously reported structure.^24^

One CRD of the manα1–4man bound structure reported here also shows the standard orientation, while the other two CRDs show the terminal sugar rotated by 180° as observed for mannose, glucosamine and fucose,^28^ and a series of disaccharides,^26^ bound to the rat MBP-C CRD. A mixture of terminal sugar orientations, observed here in one CRD (Chain B) of the manα1–4man complex, was also observed in putative structures of manα1–2man and manα1-6man soaked into trimeric MBP-A.^26^

This variation, across and within the two mannobiose structures, of ligand orientation and its medium- to long-range weak interactions with protein reflect not only the varying crystal contacts of Asp325, Gly326, Asp324, and Asn323 and their influence, via bound ligand, on Arg343 (see Fig. 4) but also the two different man–man linkages. Thus, since the same crystal contacts are present in both structures, and indeed in all the hSP-D structures derived from this crystal form,^14,17–19,24^ the linkage to the terminal sugar also appears to be a defining factor in the position and orientation of bound ligand. Nevertheless, the observed variation in manα1–4man binding orientation suggests that while a particular electronic configuration of the terminal sugar ring may be preferred, it is not a prerequisite for binding. This presumably provides an increased ability to bind a wider range of pathogens, although the strength of the interaction may vary. Significantly, the structures do not allow a definitive assignment of the preferred binding mode of this ligand and perhaps of other ligands.

For more complex ligands with suitable double hydroxyl patterns, the links between the bound sugar residue and its neighbours may, as for a terminal galactose in a physiologically relevant ligand, preclude binding. Where accessible O3′ and O4′ equivalents are present in the ligand, it seems likely that the importance of the favourable weak interactions, particularly those involving Asp325, Arg343, and the bound sugar, will be maintained with flexibility in ligand orientation if the overall ligand topology is such that the preferred binding mode cannot be achieved spatially. Despite this flexibility, affinity may be greatly reduced or abolished if there are sterically limited opportunities for overall and/or internal orientational rearrangement of ligand. In addition, while the combined structural evidence shows clearly that Asp325 is a major ligand-binding determinant, there is as yet no structural data that reveal the orientation and position of this key residue, or, as a result, bound ligand, in a physiologically relevant setting. The influence of the crystal thus hinders definitive assignment of preferred binding modes, suggesting that caution should be exercised in attempts to describe the binding of extended natural ligands based on the structurally characterised ligand-binding modes of a subset of their terminal carbohydrate substituents. Indeed, this structural feature may offer an explanation for the failure of current rfhSP-D crystals to bind ligands such as *N*-acetyl muramic acid, GalNAc-4-sulfate, and bacterial and host-genome nucleic acids and their components, which have been identified as ligands by BIAcore chip binding studies based on competition with the SP-D–mannan interaction.^3,4,9,10^

## Materials and Methods

### Crystallisation and data collection

The expression and purification of the recombinant homotrimeric fragment of hSP-D (rfhSP-D), consisting of the 177 C-terminal residues (Gly179 to Phe355), have been described previously.^30,34^ Native crystals of rfhSP-D were grown in sitting drops consisting of an equal volume (2 μl) of protein solution (7.5 mg ml^− 1^ protein in 10 mM Tris, 140 mM NaCl, and 10 mM CaCl_2_, pH 7.4) and precipitant buffer (15.75–16.5% polyethylene glycol 4000  in 100 mM Tris, pH 8.0) as described previously.^17^ IP, galactose, manα1–2man, and manα1–4man were purchased from Sigma. Crystals were soaked with ligand and prepared for cryocooling by addition of ligand and 10 mM CaCl_2_ to 2,4-methylpentane diol (MPD) cryobuffers prepared using MPD in precipitant buffer. Successive addition of 2 μl aliquots of increasing concentrations (5, 10, 15, and 20%) of MPD cryobuffer to the well was carried out, followed by addition of a further 2 μl aliquot of 20% MPD cryobuffer and an exchange of the resulting buffer with 20% MPD cryobuffer. The final concentrations of ligand in the cryobuffers were as follows: 10 mM IP, 100 mM galactose, 6 mM manα1–4man, and 10 mM manα1–2man. The IP, galactose, and manα1–4man data were collected on an ADSC Quantum 4R CCD detector on Daresbury SRS station 14.1. Low-resolution (3.0 Å, 125 images, 1.5° oscillation) and high-resolution (200 images, 1.0° oscillation) data sets were collected for galactose and manα1–4man ligands. The manα1–2man data were collected on the microfocus beamline ID13 at the European Synchrotron Radiation Facility. The crystal was of maximum dimension 50 μm, and data were collected in three segments with successive translations to a fresh part of the crystal for each data segment. Integrated intensities were calculated with the program MOSFLM.^38^ Data collection and processing statistics are given in Table 3.

### Structure solution and refinement

Isomorphism was sufficient to allow the coordinates of the previously determined 1.6-Å rfhSP-D structure^17^ to be used as a starting model for the ligand-bound rfhSP-D structures. The structures were refined using the programs X-PLOR^39^ (initially) and CNS.^40^ Maps were calculated using CNS and the CCP4 program suite,^41^ including density modification (solvent flattening and histogram matching, but not NCS averaging), and models were built using the graphics program O.^42^ Topology and parameter files for ligand were obtained from the HIC-Up server.^43^ Refinement statistics are given in Table 3. All main- and side-chain stereochemical parameters either fall inside or are better than those expected (PROCHECK^44^) with no residues in generously allowed or disallowed regions. Molecular figures were generated using MOLSCRIPT.^45^

### Accession numbers

The coordinates and structure factors for manα1–2man (3IKQ), manα1–4man (3IKR), galactose (3IKN), and IP (3IKP)-bound rfhSP-D have been deposited with the PDB.

## Figures and Tables

**Table 1:** Calcium and ligand binding

			Chain A				Chain B				Chain C			
Atom 1	Atom 2		gal	m12m	IP	m14m	gal	m12m	IP	m14m	gal	m12m	IP	m14m
Ca1	Glu321	OE1	2.69	2.41	2.57	2.63	2.62	2.62	2.55	2.50	2.58	2.62	2.56	2.49
	Asn323	OD1	2.62	2.57	2.48	2.50	2.52	2.38	2.42	2.38	2.54	2.47	2.41	2.40
	Glu329	OE1	2.52	2.34	2.49	2.44	2.57	2.40	2.49	2.46	2.53	2.31	2.36	2.46
	Asn341	OD1	2.55	2.51	2.38	2.40	2.48	2.47	2.34	2.38	2.49	2.57	2.38	2.38
	Asp342	OD1	2.48	2.31	2.28	2.33	2.46	2.22	2.24	2.30	2.46	2.29	2.25	2.29
	Asp342	O	2.66	2.66	2.62	2.63	2.64	2.63	2.58	2.57	2.67	2.66	2.59	2.61

	Ligand	O_a_	2.60	2.78	2.59	2.52	2.59	2.44	2.58	2.56	2.54	2.47	2.57	2.53
		O_b_	2.66	2.58	2.49	2.55	2.61	2.86	2.54	2.58	2.67	2.55	2.55	2.66

	Ligand	W			2.59									
		W			2.49									
		O1′	2.66				2.61				2.67			
		O2′	2.60				2.59				2.54			
		O3′		2.78		2.52		2.44		2.58		2.47		2.66
		O4′		2.58		2.55		2.86	2.58	2.56		2.55	2.57	2.53
		O5′							2.54				2.55	

O_a_	Glu321	OE2	2.67	2.49	2.69	2.58	2.55	2.56	2.56	2.61	2.60	2.40	2.58	2.62
	Asn323	ND2	2.73	2.86	2.65	2.67	2.86	2.84	2.91	2.86	2.96	3.01	2.84	2.94
O_b_	Glu329	OE2	2.69	2.52	2.62	2.63	2.56	2.51	2.59	2.45	2.58	2.42	2.62	2.55
	Asn341	ND2	2.98	3.20	3.02	2.95	3.00	3.12	2.96	3.07	3.05	3.29	3.02	3.02

The galactose-bound structure is indicated by gal, the α1-2 mannobiose by m12m, the inositol phosphate by IP, and the α1-4 mannobiose by m14m. As shown in subsequent rows of the table, O_a_ and O_b_ represent O2′ and O1′ in the galactose-bound structure, O3′ and O4′ for α1-2 mannobiose and for chain A of the α1-4 mannobiose structure, O4′ and O3′ in chains B and C of the α1-4 mannobiose structure, O4′ and O5′ in chains B and C of the inositol phosphate structure, and water molecules in chain A of the inositol phosphate structure. All distances are given in angstroms.

**Table 2:** Protein–protein symmetry contacts in the vicinity of the ligand-binding site and protein–ligand interactions involving Arg343 and Asp325

Molecule 1		Chain	Symmetry molecule 2		Chain	nat	malt	gal	IP	m12m	m14m
Asp325	OD1	A	Tyr314	OH	B	2.63	2.75	2.77	2.58	2.66	2.61
Asp325	OD1	B	Lys230	NZ	C	2.85	2.85	2.77	2.79	2.99	2.80
Asp325	OD1	C	Lys230	NZ	A	3.00	3.03	2.89	3.14	3.24	2.85
Gly326	N	A	Asn316	OD1	B	2.97	2.84	2.85	2.84	2.91	2.82
Asn323	O	A	Lys339	NZ	B	2.80	2.79	2.80	2.73	2.95	2.68
Asp324	O	A	Lys339	NZ	B	3.09	2.96	2.99	2.91	3.30	2.97

Asn323	ND2	B	Ser226	OG	C	2.85	2.88	2.90	2.80	3.20	2.87
Asn323	ND2	C	Ser226	OG	A	3.02	3.09	3.10	3.06	3.13	2.95

Pro322	O	C	Gln222	NE2	A	3.08	2.99	2.83	2.82	3.10	2.99
Pro322	O	B	Gln222	NE2	C	2.99	2.98	2.99	2.99	2.98	3.01

Protein		Chain	Ligand		Sugar	nat	malt	gal	IP	m12m	m14m

Arg343	NH2	A	Maltose	O6′	glc1		3.06				
	NH1	B		O2′			3.5				
	NH2	C		O6′			3.34				
	NH1	A	Galactose	O6′	gal			3.13			
	NH1	A		O5′				3.24			
	NH2	A		O5′				3.19			
	NH1	B	Galactose	O6′	gal			2.97			
	NH1	B		O5′				3.10			
	NH2	B		O5′				3.08			
	NH1	C	Galactose	O6′	gal			2.92			
	NH1	C		O5′				2.98			
	NH2	C		O5′				3.06			
	NE	B	IP	O6′	I				2.93		
	NE	C		O6′					3.20		
	NH1	A	m12m	O6′	man1					2.87	
	NH2	A	m14m	O6′	man1						3.34
	NH1	B	m14m	O2′	man1						3.20
	NH1	C	m14m	O2′	man1						3.29
Asp325	OD2	B	m12m	O6′	man1					2.85	
	OD2	B	m14m	O1′	man1						3.39
	OD2	C	m14m	O1′	man1						3.22

The unliganded and maltose-bound structures are indicated by nat and malt, respectively,^17^ galactose by gal, inositol phosphate by IP, manα1–2man by m12m, and manα1–4man by m14m. Glc1 and man1 represent the terminal sugar of the bound disaccharides maltose and mannobiose, respectively. The three chains in the rfhSP-D trimer are shown by A, B, and C. All distances are given in angstroms.

**Table 3:** Crystallographic data and refinement statistics for rfhSP-D ligand soaks

	Galactose	manα1–4man	IP	manα1–2man
*Data collection*				
Wavelength (Å)	1.488	1.488	1.488	0.954
Temperature (K)	100	100	100	100
Space group	*P*2_1_	*P*2_1_	*P*2_1_	*P*2_1_
Cell dimensions				
*a* (Å)	55.74	55.52	55.45	56.08
*b* (Å)	108.58	108.45	107.72	108.78
*c* (Å)	55.88	55.82	55.67	55.91
β (°)	91.41	91.25	91.23	89.81
Maximal resolution (Å)	1.6	1.65	1.75	2.25
Highest-resolution bin (Å)	1.69–1.60	1.74–1.65	1.84–1.75	2.37–2.25
Observations	354,154 (23,390)	353,449 (36,056)	227,295 (24,386)	106,719 (9044)
Unique reflections	84,773 (8032)	74,691 (9255)	64,893 (7824)	31,502 (3256)
Completeness (%)	95.8 (84.6)	94.5 (87.7)	98.7 (94.3)	99.0 (97.4)
*R*_merge_^a^	0.040 (0.119)	0.037 (0.151)	0.077 (0.194)	0.059 (0.222)
*I*/σ(*I*)	9.4 (4.4)	11.1 (4.3)	4.2 (3.3)	9.5 (1.6)

* Refinement*				
Protein atoms^b^	3483	3476	3464	3336
Residues, chain A	204–355	204–355	205–355	210–355
Residues, chain B	204–355	205–355	204–355	210–355
Residues, chain C	204–355	204–355	206–355	211–355
Other atoms				
Calcium ions	10	10	10	10
Ligand	36	36	32	36
Water	492	519	440	182
Resolution range (Å)	55.5–1.60	39.8–1.65	55.0–1.75	30.4–2.25
*R*_conv_^c^ (%)	20.1	19.3	19.8	20.2
*R*_free_^d^ (%)	21.5	21.2	21.3	23.9
Average *B*-values (Å^2^)				
Protein main chain	21.0	18.6	21.7	25.0
Ligand	23.2	27.1	31.0	38.0
Water	31.1	29.5	33.3	29.1
Ramachandran plot values^e^ (%)				
Most favoured	92.6	93.5	94.1	92.4
Additional allowed	7.4	6.5	5.9	7.6
Disallowed	0.0	0.0	0.0	0.0

Figures in parentheses refer to the highest-resolution bin.

a *R*_merge_ = ∑*_h_*∑*_j_*∣*I_h_*_,*j*_ − *I*_*h*_∣/∑*_h_*∑*_j_*∣*I*_*h*__,*j*_∣, where *I_h_*_,*j*_ is the *j*th observation of reflection *h* and *I*_*h*_ is the mean of the *j* measurements of reflection *h*.

b Excluding alternative side-chain conformations.

c *R*_conv_ = ∑*_h_*∥*F*_o*h*_∣ − ∣*F*_c*h*_∥/∑*_h_*∣*F*_o*h*_∣, where *F*_o*h*_ and *F*_c*h*_ are the observed and calculated structure factor amplitudes, respectively, for reflection *h*.

d *R*_free_ is equivalent to *R*_conv_ for a 5% subset of reflections not used in the refinement.

e Defined according to PROCHECK.
